# SURVEILLANCE PROGRAM IDENTIFIES HEALTH OUTCOMES FOR FORMER WORKERS

**DOI:** 10.1093/geroni/igz038.3450

**Published:** 2019-11-08

**Authors:** Ashley Golden, Jeffery Miller, Zachariah Hubbell, Sara Howard, Donna Cragle

**Affiliations:** 1 Oak Ridge Associated Universities, Oak Ridge, Tennessee, United States; 2 Oak Ridge Associated Universities, Arvada, Colorado, United States

## Abstract

According to the US Bureau of Labor Statistics, the average age of retirement is 62. While many retirees may have employer-provided or other access to healthcare, others have limited access to affordable care until full Medicare eligibility at 65. Regardless of access, retirees with toxic occupational exposures may not have providers with specialized knowledge of tests or diagnoses for exposure-related health conditions, especially those with long-latency. The National Supplemental Screening Program for U.S. Department of Energy Former Workers is described here as a nationwide program providing recurring (every 3 years) integrated health screenings designed to identify both occupational and non-occupational conditions in the context of exposure so that early identification can enable appropriate and timely diagnoses and treatments to improve health outcomes. Since September 2005, there has been 18,518 initial exams for former workers, of whom 5,461 returned for rescreening exams through April 2019. The average age of those returning was significantly younger at initial exam (63.4 years) compared to those who did not return (65.1 years). The most common occupational condition was noise-induced hearing loss not attributable to natural, age-related loss (67%). Rare and long-latency occupational health conditions, such as asbestosis or silicosis, were identified at rates expected (1-4%). The most common non-occupational condition was elevated body mass index (BMI>25, 77.3%), followed by hypertension (20.7%), of which 50% had no prior knowledge or clinical diagnosis. In conclusion, occupational health surveillance programs can provide value for identifying non-occupational health conditions and as a supplementary source of health information and care.

